# Implications of CTL-Mediated Killing of HIV-Infected Cells during the Non-Productive Stage of Infection

**DOI:** 10.1371/journal.pone.0016468

**Published:** 2011-02-07

**Authors:** Christian L. Althaus, Rob J. De Boer

**Affiliations:** Theoretical Biology, Utrecht University, Utrecht, The Netherlands; University of Palermo, Italy

## Abstract

Patients infected with HIV exhibit orders of magnitude differences in their set-point levels of the plasma viral load. As to what extent this variation is due to differences in the efficacy of the cytotoxic T lymphocyte (CTL) response in these patients is unclear. Several studies have shown that HIV-infected CD4^+^ T cells also present viral epitopes that are recognized by CTLs before the productive stage of infection, i.e., during the intracellular eclipse phase before the infected cell starts to produce new viral particles. Here, we use mathematical modeling to investigate the potential impact of early killing of HIV-infected cells on viral replication. We suggest that the majority of CTL-mediated killing could occur during the viral eclipse phase, and that the killing of virus-producing cells could be substantially lower at later stages due to MHC-I-down-regulation. Such a mechanism is in agreement with several experimental observations that include CD8^+^ T cell depletion and antiretroviral drug treatment. This indicates a potentially important role of CTL-mediated killing during the non-productive stage of HIV-infected cells.

## Introduction

Infection with HIV typically leads to a vast replication of the virus during the acute phase of the infection which is followed by a chronic phase where the viral load approaches a quasi-steady-state, known as the viral set-point. It has been shown that the viral load levels can vary over orders of magnitude between patients [1]–[3] and the set-point level has been recognized to be an important predictor for disease progression [4]. Part of the difference in the control of HIV replication between patients has been attributed to varying efficacies of the patient's immune responses to induce cytotoxic T lymphocyte (CTL)-mediated killing of infected cells [5]–[8]. A major role of the CD8^+^ T cell response in controlling HIV infection is further supported by the very rapid evolution of immune escape variants during the first months of infection [9]–[11]. However, it is remarkable that the virus load declines at very similar rates in different patients when they are treated with antiretroviral drugs [12], [13]. The viral load decay during antiretroviral therapy is typically related to the loss of HIV-infected cells and occurs at a rate between 0.5 and 1.5 per day [14]. Recent experiments with depleting CD8^+^ T cells by antibodies have further indicated that the rate at which virus-producing cells are cleared during antiretroviral therapy is unaffected by the presence or absence of CD8^+^ T cells [15], [16]. Therefore, it is puzzling how CTLs could account for large differences in the viral set-point causing a controversy as to whether CTLs mediate control of HIV through cytotoxic or non-cytotoxic mechanisms [17]–[19].

Klenerman et al. [20] have presented a mathematical model to show that CTLs can markedly reduce the virus load by limiting virus production with minor effects on the half-life of infected cells. Assuming that the rate at which an infected cell becomes a target for CTLs is slow (e.g. 0.4 d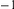), it will be this transition rate rather than the death rate of the cells that is reflected in the viral load decline [20]. Others have adopted this model in combination with experiments to highlight the impact of epitope expression kinetics on the recognition of HIV-infected cells by CTLs [21], [22]. Newer studies, however, have shown that HIV-infected cells become a target for CTLs as soon as 2 to 6 hours after infection [23]–[25]. It was further shown that SIV-specific CD4^+^ T cells recognize and inhibit viral replication very early after infection of a cell [26]. This suggests that the transition rate at which cells become recognized and turn into a target for CTLs is very fast (4 to 12 d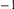), and is much higher than the typical decline rate of viral load after drug treatment. This observation casts doubt on the explanation of Klenerman et al. [20], and highlights two important new features. First, that infected cells become a target for CTL-mediated killing very early, and second, that infected cells can be killed during the non-productive stage of infection, i.e., during the intracellular eclipse phase.

Interestingly, HIV evolved a mechanism to partially evade killing by CTLs through down-regulation of MHC-I molecules in infected cells [27]–[29]. Down-regulation is induced by the protein Nef [30] and starts as early as 12 h post-infection [24]. The intracellular eclipse phase lasts around 24 hours [31]–[33]. Hence, CTL-mediated killing of infected cells during the eclipse phase can be more efficient because MHC-I is not yet down-regulated. CTLs recognizing epitopes that are presented very early, such as epitopes derived from the viral protein Gag, might thus mediate efficient cytotoxic killing soon after the cell has become infected. In contrast, virus-producing cells that partly evade killing by CTLs by MHC down-regulation, are expected to be killed by CTLs, natural killer (NK) cells, and the cytopathic effects of viral production, which in combination would have to account for the typical death rate of infected cells (0.5–1.5 d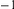) that is observed after the start of drug therapy. The fact that the death rate of infected cells that are actively producing SIV is hardly decreased when CD8^+^ CTL and NK cells are depleted [15], [16], seems to suggest that this typical death rate is largely determined by the cytopathic effects of viral production.

We devise a mathematical model of HIV dynamics to investigate the impact of CTL-mediated killing of HIV-infected cells during the intracellular eclipse phase. Our model can account for large variations in viral set-point levels without affecting the typical decay rate of virus load after drug treatment. We further test the consistency of the hypothesis that HIV-infected cells are cleared during the non-productive stage of infection with several experimental observations such as the unexpected effects of CD8^+^ depletion experiments during antiretroviral therapy [15], [16]. While non-cytotoxic mechanisms of CD8^+^ T cells cannot be ruled out, our study indicates a potentially important role of cytotoxic killing of HIV-infected cells during the intracellular eclipse phase.

## Results

### Early vs. late killing of HIV-infected cells

**Figure 1:**
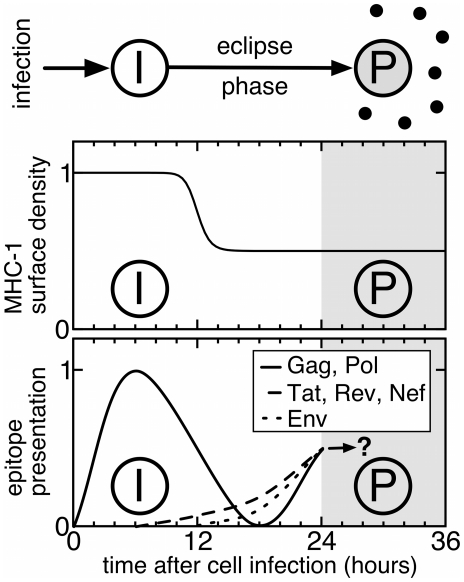
MHC-I density and epitope presentation on the surface of an HIV-infected cell. On top, an infected cell, 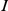, is shown that passes through the eclipse phase to become a virus-producing cell, 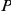. In the middle panel, the relative MHC-I density is shown as being down-regulated around 12 h after infection. The bottom panel depicts the early surface presentation of epitopes during the first 24 hours after infection. Gag and Pol epitopes show a peak shortly after infection since they are derived from the infecting virus particles. Later, *de novo* synthesis of viral proteins takes place, generating the early proteins Tat, Rev and Nef and the late proteins Env, Gag and Pol. The viral eclipse phase is denoted by the white area. After the eclipse phase, virus-producing cells, 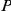, are likely to experience a moderate death rate due to the MHC-I-down-regulation (gray area). The illustrations are based on kinetic data from Sacha et al. [23], [24].

Our model considers two types of cells infected with HIV. Infected cells during the viral eclipse phase, 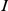, do not produce virus yet, whereas cells that pass the eclipse phase (caused by an intracellular delay of virion production) become productively infected cells, 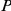, and release new viral particles (Fig. 1, top). Both populations experience cell death, at a rate 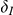 and 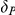 respectively, that can at least partly be due to CTL-mediated killing (see *Methods* for the mathematical description).

**Figure 2:**
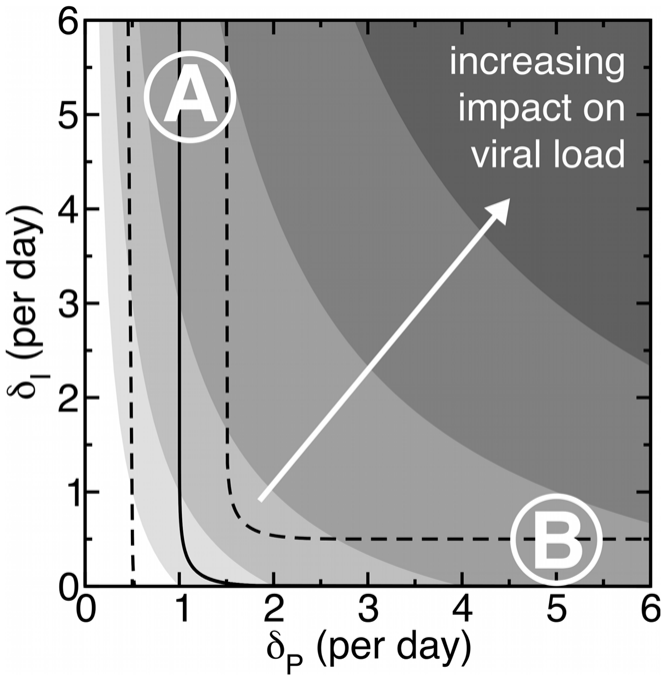
Influence of early and late death rates of HIV-infected cells on the viral load and the slope of the virus decline after drug treatment. During the viral eclipse phase, infected cells, 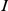, experience a death rate of 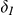 per day whereas virus-producing cells, 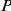, die at a rate 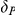. The contour lines depict the impact of cell death on reducing the viral load (see *Methods*). The area where the virus decline slope is between 0.5 d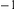 and 1.5 d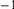 is given by the dashed lines. Region A: Infected cells experience a higher death rate during the eclipse phase than later when they start to produce new viral particles. Hence, the slope of the virus decline after drug treatment is determined by 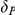. Region B: As the death rate of infected cells during the eclipse phase is low, the decline slope after drug treatment is determined by the rate at which cells move through the eclipse phase (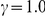 d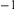). The exponential slope of the virus decline is calculated at 5 days after the start of treatment. For the mathematical derivation we refer to *Methods*.

First, we analyze which parameter regimes realistically describe the characteristic early down slope of 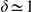 d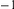 of the viral load decay during effective antiretroviral treatment [12]–[14]. All parameter combinations in the area between dashed lines in Fig. 2 would account for a realistic downslope of 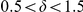 d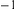 (see *Methods* for how these lines are computed). The influence of the death rates of infected cells, 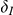, and virus-producing cells, 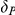, on the reduction of the viral set-point is indicated by the contour lines in Fig. 2. One realistic possibility to reduce the viral load significantly is that virus producing cells die rapidly, i.e. 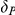 is high, while cells in the viral eclipse phase die slowly, i.e. have a low 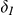, and have an eclipse phase of approximately one day (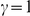 d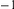). This is depicted by region B in Fig. 2, and corresponds to the regime previously described by Klenerman et al. [20]. In this regime the decline slope after drug treatment is reflecting the rate at which cells move through the eclipse phase (i.e., 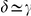 in the model from Eq. 1). Our main result is the new regime depicted by region A in Fig. 2 with early killing of infected cells during the intracellular eclipse phase, i.e., high 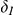. This falls in the realistic area when the death rate of producing cells would indeed correspond to the observed downslope of the viral load during treatment (i.e., 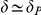). The length of the eclipse phase is hardly reflected in the downslope 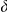 in the ‘early killing’ regime. Note that there is no realistic regime where both early and late killing could be fast. The new evidence for early killing [23]–[25] provides support for the new regime depicted by region A in Fig. 2. In this paper we investigate the implications of killing mediated early during the intracellular eclipse as the main mechanism at which HIV-infected cells are controlled by CTLs.

### Set-point viral load

To explicitly analyze the effect of early CTL-mediated killing of HIV-infected cells on the set-point viral load, we describe the death rate of infected cells as 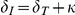, which sums the natural death rate of CD4^+^ cells, 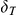, with a function describing CTL-mediated killing, 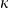. The death rate of virus-producing cells, 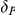, remains small and constant and is a combination of natural death, virus induced cytotoxicity, moderate CTL-mediated killing, and killing by NK cells as a consequence of MHC-I down-regulation.

**Figure 3:**
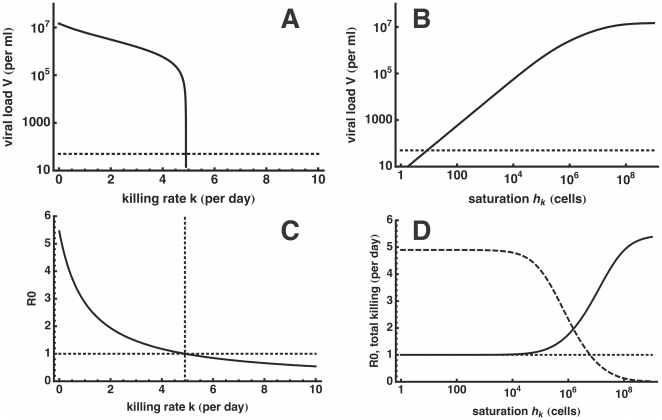
Set-point viral load and the basic reproductive number, 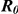, as a function of CTL-mediated killing. The left panels depict the behavior when killing is induced with a total rate 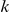 per day. The viral load decreases sharply for 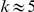 d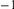 (A) since 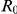 falls below one, and the infection is cleared (C). If CTL effector cells form a complex with the infected cell before delivering their lethal hit, we assume a maximal killing rate (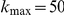 d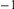) and change the Michaelis-Menten constant 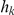 instead. This way, we can account for orders of magnitude differences in the viral load (B) since 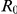 is slowly approaching one (solid line, D). The total killing (dashed line, D) induced by all CTL effector cells is approaching the same critical value around 5 d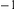 although 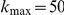 d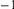. In panel (A) and (B), the dotted line represents the usual detection limit for HIV-1. The dotted line in panel (C) and (D) depicts the 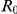 of 1 below which the infection cannot sustain itself anymore.

First, consider the simple case with a constant CTL response, i.e. 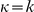, where the death rate during the eclipse phase, 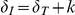, is a constant like in Fig. 2. Here the total CTL response kills infected cells, 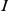, at a rate 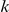 per day. Increasing 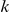 reduces the set-point viral load (Fig. 3A), and increasing the rate of killing above a threshold of 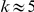 d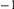 will clear the infection. The basic reproductive number (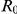) indeed falls below 1 at the same critical value of 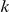 (Fig. 3C, see *Methods* for the definition of 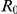). Early killing can therefore be extremely efficient, and could in theory clear the infection if infected cells during the eclipse phase are expected to be killed at a rate exceeding a value of approximately 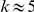 d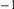, i.e. would on average be killed in about five hours after infection.

Second, consider CTL effector cells 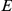 that proliferate according to a function 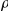 and die at a rate 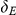 per day. These CTLs kill infected cells, 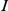, according to a specific function 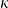. For the detailed mathematical description of the CTL proliferation 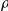 and the CTL-mediated killing 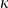, we refer to *Methods*. To account for different efficacies of CTL-mediated killing we can change the saturation parameter of the killing term, 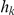, in this function. 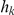 is a generalized Michaelis-Menten constant, and decreasing 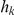 increases the killing rate of cells expressing ‘early’ epitopes. This allows us to have a single parameter defining the ‘immune responsiveness’, which turns out to account for orders of magnitude differences in viral load set-point levels (Fig. 3B). The corresponding 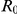 now approaches 1 when 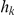 is reduced, i.e., the efficacy of CTL-mediated killing is increased to 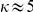 d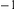 (Fig. 3D).

This analysis demonstrates that ‘early killing’ is an efficient control mechanism, i.e., can account for large variations in the viral load between patients by substantial differences in the efficacy of the immune response, and for a critical killing rate at which the infection is cleared. However, it does not imply that early killing is more efficient than late killing, or that the critical killing rate of approximately 5 d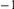 is a reliable estimate. First, the killing rate at which the infection is cleared does depend on other parameters (not shown). But tuning the other parameter values such that we obtain a realistic viral replication rate of 1.5 d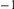 during the acute phase of infection (see Table 1) results in critical killing rate that is at least plausible. Second, one can also repeat the bifurcation analysis of Fig. 3 for different assumptions on the distribution of the killing over the early and the late stages of an infected cell. One possibility is that most killing occurs late, which we can study by letting 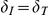 and 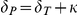. This yields a very similar critical rate of about 5.5 d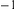 (not shown). Late killing is therefore an equally efficient control mechanism, and would be consistent with the characteristic early down slope of 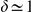 d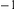 of the viral load decay during effective antiretroviral treatment [12]–[14] when there is little early killing, and an eclipse phase of approximately one day (see region B in Fig. 2) [20]. Alternatively, the killing rates during the early and late phases could be assumed to be similar, e.g., by setting 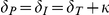. This results in a lower but still relatively high threshold killing rate of approximately 2 d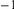 (not shown). Killing that is mediated during the early and late stage of an infected cell is therefore also an efficient control mechanism but would require a viral load decay during antiretroviral treatment that is not consistent with the characteristic down slope of 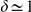 d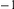 [12]–[14].

**Table 1:** List of parameter values for the HIV dynamics model.

Parameter	Value	Explanation and reference
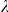	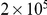 cells d 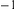	Tuned to obtain a maximal viral load around 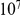 per ml in the absence of a CTL response.
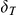	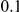 d 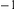	Natural death rate of CD4^+^ target cells 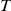 .
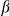	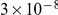 d 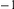	Infection rate per virus particle. Results in a maximal viral replication rate of 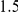 d 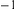 [61].
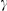	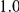 d 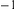	Viral eclipse phase of 24 hours [31], [32].
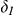	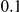 d 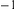	Natural death rate of infected cells 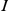 .
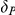	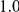 d 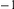	Death rate of virus-producing cells [31], [32].
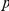	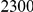 d 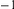	Virus production rate.
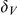	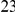 d 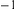	Clearance rate of viral particles [56].
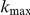	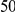 d 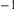	Maximal killing rate [62].
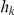	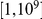 cells	Variable efficacy of CTL response.
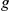	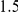 d 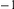	Maximal CTL proliferation rate of 1.0 d 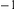 [63].
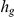	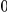 cells	CTLs are stimulated to proliferate rapidly.
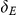	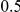 d 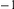	Death rate of CTL effector cells.

Summarizing, we have shown that if CTL-mediated killing happens before the infected cell starts to produce new viral particles, the viral load can be suppressed very effectively by the CTL response, independent of the predicted downslope of the viral load during drug treatment. The infection can be cleared when the killing rate during the eclipse phase exceeds a rate of 5 per day. Such a high killing rate has never been consistent with the viral load decay data, and has therefore has not been considered in studies of the effect of CTL on controlling HIV-1 infection [34].

### Reproducing experimental observations of HIV dynamics

#### Viral load decay during drug treatment

**Figure 4:**
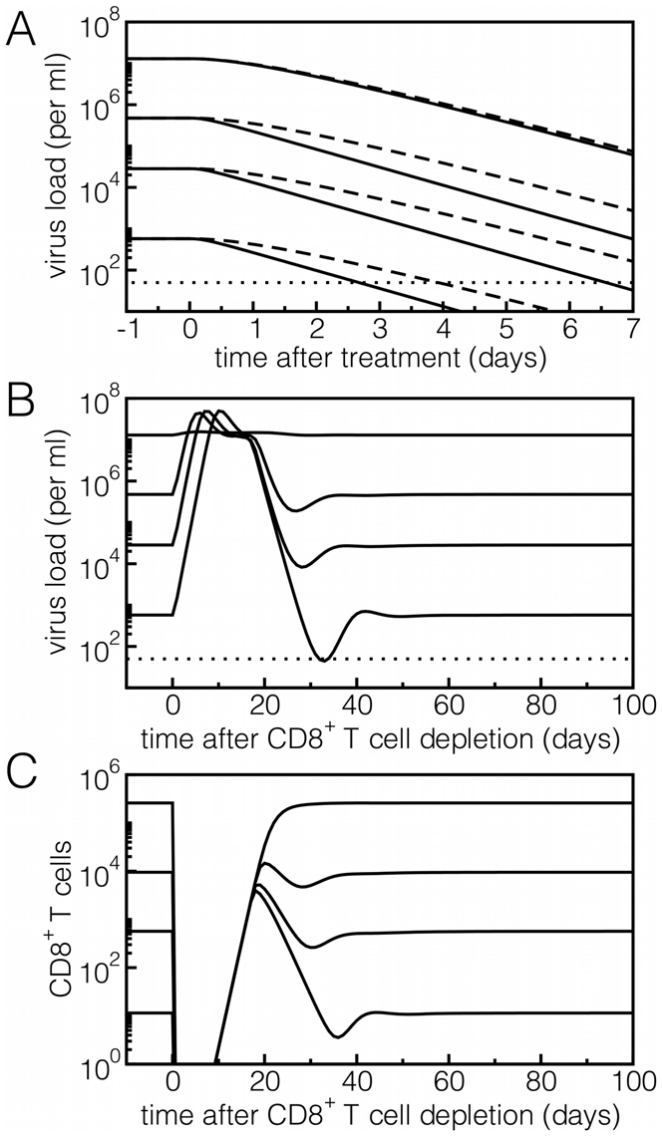
Reproducing experimental observations of HIV dynamics. A) After drug treatment, the viral load declines proportionally to the number of virus-producing cells (straight lines). Since the death rate of these cells, 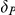, is constant, we observe the same slope for the exponential decay of viral load although different patients have varying efficacies of their CTL response. Hence, the dynamics is not affected by the killing efficacy 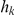 which determines the set-point level of viral load before treatment. If CTLs were depleted at the start of drug treatment, the same phenomenon would be observed. The virus load declines exponentially with the death rate of virus-producing cells that is unaffected by CTL-mediated killing (dashed lines). B) In CD8^+^ depletion experiments, the viral load increases over orders of magnitude, peaks around two weeks after depletion, reduces again, and stabilizes afterwards at the previous set-point level. C) The CTLs are depleted and start to proliferate thereafter. Black lines denote different hypothetical patients with varying efficacies of their CTL response. Lines from top to bottom: 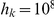, 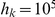, 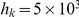 and 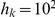. After CD8^+^ depletion, the concentration of CTL effector cells is set to 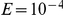. The dotted lines represent the usual detection limit for HIV-1.

We demonstrated above that ‘early killing’ can account for large differences in viral set-point levels while the observed death rate of virus-producing cells during antiretroviral therapy would remain largely invariant. This characteristic behavior is depicted by the solid lines in Fig. 4A, where the set-point viral load varies over orders of magnitude when the saturation constant of the immune response is changed, but decreases with the same rate after starting therapy. Recent experiments combining drug treatment with CD8^+^ T cell depletion also suggested that CTL-mediated killing is not responsible for the death of virus-producing cells. In SIV-infected rhesus macaques, the viral load decay following the administration of antiretroviral drugs was not different in CD8^+^ T depleted animals compared to controls [15], [16]. In our model we can reproduce this observation by performing an *in silico* experiment where CD8^+^ depletion takes place at the same time when drug treatment is started. Technically speaking, we set the infection rate 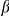 to zero and the number of CTL effector cells to low levels at the onset of treatment. Because in our ‘early killing’ regime the death rate of virus producing cells hardly depends on the CTL response, we obtain the same exponential decay of the viral load as observed when CTL are not removed (dashed lines in Fig. 4A). Interestingly, the shoulder phase after the start of treatment, that is typically assumed to be caused by the intracellular delay, becomes longer when CD8^+^ depletion is performed. This is due to the fact that a larger fraction of the cells in the eclipse phase at the start of treatment will move into the state of virus-producing cells when CTLs are removed.

#### Early CD8^+^ depletion experiments

Strong evidence that CTLs are responsible in suppressing the viral load during chronic HIV infection comes from the marked increase in the viral load after CD8^+^ T cell depletion. Monkeys infected with simian immunodeficiency virus (SIV) show a rapid and transient increase in their viral load over orders of magnitude upon depleting their CD8^+^ T cells [6], [35], [36]. This observation was difficult to explain with a mathematical model [35] in which the CD8^+^ T cell depletion affects the death rate of virus-producing cells. Jin et al. [35] also investigated non-cytotoxic mechanisms, such as increasing virus production or increasing infection rates, but concluded that the model fails to accurately describe the experiments. Later, it was suggested that an ‘early cytotoxic’ immune response could eliminate infected cells before they produce viral particles, and such a model readily explained the large and rapid increase in the viral load in the CD8^+^ T cell depletion experiments [2]. Similarly, with our model that explicitly takes into account a CTL response targeting infected cells during the intracellular eclipse phase, we can account for a strong suppression of virus replication during the chronic phase of infection. Upon CD8^+^ T cell depletion, the number of cells becoming virus-producing cells increases rapidly leading to a transient increase in the viral load (Fig. 4B). We obtain similar peak viral loads for the different set-point levels because in the absence of an immune response, the viral load in our model is expected to approach the steady-state determined by limited target cell availability, which is independent of the 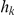 parameter. These similar levels are not in agreement with the variation in peak viral loads in the study of Jin et al. [35], which could be due to the differences in target cell availability in the CD8^+^ T cell depleted monkeys, and/or due to different efficacies of CD8^+^ T cell depletion in the monkeys. After the peak, the viral load reaches the previous set-point since the CD8^+^ T cells start to proliferate again (Fig. 4C). Thus, the rapid and dramatic effects of CD8^+^ T cell depletion are perfectly consistent with cytotoxic control of viral replication, if – and only if – this control occurs early in the life cycle of an infected cell.

## Discussion

The traditional concept of recognition of HIV-infected cells by CTLs was that the cells have to express viral proteins first before they can present viral epitopes on their surface. This belief implied a puzzling problem because specific CD8^+^ T cell responses appear to be strongly associated with different viral set-point levels in patients [3], [37], but the death rate of virus-producing cells is very similar in different patients [14]. The notion that the death rate of virus-producing cells is largely unaffected by the CTL response was further supported by the similar decay kinetics of the viral load in treated natural hosts of SIV, i.e., sooty mangabeys and African green monkeys, that experience little immune activation and trigger weak CTL responses [38], [39]. This problem raised questions about the role that CTL play in controlling HIV-1 infection. In this paper we have shown that these seemingly contradictory findings become perfectly consistent when most of the CTL-mediated killing of HIV-infected cells occurs during the viral eclipse phase, i.e., during the intracellular delay before the infected cell starts to produce new viral particles. During the viral eclipse phase, HIV-infected cells can be recognized by epitopes derived from the proteins that enter the cell with a viral particle [23]–[26].

Another observation that caused controvery on the role of CTL in controlling HIV-1 infection was the absence of an effect of depleting CD8^+^ T cells on the death rate of virus-producing cells [15], [16]. One interpretation of these experiments was that specific CD8^+^ T cells largely exert non-cytotoxic effects (while it was also stated that the possibility of CTL-mediated killing during the non-productive stage of infection cannot be ruled out). We have shown that cytotoxicity during the eclipse phase is indeed perfectly consistent with the absence of an affect of CD8^+^ T cell depletion during antiretroviral treatment. An experiment to test whether CTL-mediated killing acts during the intracellular eclipse phase would be to follow the dynamics of different cell populations after drug treatment. The virus-producing cells are expected to decline with the same rate as the viral load. In contrast, we hypothesize that infected cells during the early stage before MHC-I-down-regulation experience strong CTL-mediated killing, and therefore should decay with a faster rate than the viral load.

The intriguing implication of the studies from Sacha et al. [23]–[26] is that they provide an explanation why CTL responses targeting epitopes from the protein Gag are more efficient in controlling the viral replication than CTL responses targeting epitopes from other viral proteins [3], [40]. Since Gag epitopes are presented on the infected cells surface early after infection, the CTLs have a time-window of about ten hours to recognize infected cells before their MHC-I is down-regulated. Although Pol, Vpr and Rev epitopes are presented early as well, responses targeting those epitopes seem to be less efficient in controlling the viral replication. It has been argued that the number of proteins in a virion that enter the cell leads to a different concentration of protein-derived epitopes on the cell surface. While Gag proteins are highly abundant, with a copy number of 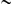
5000 per virion [41], Pol, Vpr and Rev proteins are present at much lower copy numbers. Further, structural constraints in the protein Gag make it more difficult for the virus to accumulate epitope escape mutations in order to evade the CTL responses [40]. This could explain why the rapid escape of HIV during the acute phase of infection also occurs in epitopes derived from other proteins such as Tat and Vif [10], [11]. Because at least some of these epitopes are not expressed during the viral eclipse phase [25], specific CD8^+^ T cells also seem to play an important role during the later productive stage of an infected cell (at least during the acute phase of infection). Whether or not this occurs mainly through late killing in combination with a slow eclipse phase [20], or is largely due to non-cytolytic effects during the late productive phase of an infected cell [2], [15]–[19], [42], remains to be established.

Nevertheless, the studies by Sacha et al. [23]–[26] are in conflict with previous findings on the impact of epitope expression kinetics on the efficacy of CTL responses [43]. It has been argued before that since Gag is a ‘late’ protein that is expressed at the end of the viral eclipse phase, CTL responses against ‘early’ proteins such as Rev should be more effective in the control of the viral replication. Indeed, it was shown with recombinant viruses that RT- and Gag-specific CTL responses become much more effective in the control of virus replication if the epitopes are expressed as part of an early protein, such as Rev or Nef [21], [44]. This is in contrast to the studies by Sacha et al. [23]–[26] where epitopes from Gag, Pol and Rev were all presented early after infection at about the same time, suggesting that the efficacy of those CTL responses should not strictly correlate with the *de novo* protein expression kinetics. One possible explanation for this apparent discrepancy comes from the observation that not all epitopes from a specific protein are presented with the same kinetics, i.e., epitopes derived from the same viral protein experience differential antigen presentation kinetics [45]. Indeed, different epitopes were used in these studies [21], [23], [24], [44], which might explain the different results of their experiments. The antigen presentation kinetics of epitopes could be influenced by different cleavage efficacies of the peptides by the proteasome. Early after infection of a cell, the peptides are most likely to be cleaved by the constitutive proteasome. At a later stage, the immunoproteasome is expected to become expressed, which has a different specificity [46]. Analysis of the cleavage scores obtained by the epitope predictor NetChop [47], [48] did not result in a clearly distinct cleavage pattern between the epitopes that have been investigated in the studies referenced above (results not shown). In addition, the studies also used different assays to investigate the impact of the CTL response. In Sacha et al. [23]–[25], the elimination of p27 positive cells was followed during a single infection cycle of SIVmac239, whereas the earlier studies measured after several generations of HIV-1 infection the relative amount of p24 antigen that was suppressed by adding specific CTLs [21], [44]. Finally, the epitope presentation kinetics is only one of a magnitude of factors that determine the efficacy of CTLs [49].

Lastly, it needs to be investigated how efficient an HIV-infected cell can escape recognition by CTLs through down-regulation of MHC-I [50]. While the viral protein Nef can down-modulate HLA-A and HLA-B, the surface presentation of HLA-C is hardly affected. It has been speculated that this might be a strategy of the virus to escape the most efficient CTL responses, which are directed against epitopes presented on HLA-A and HLA-B, whereas the lack of down-regulation of HLA-C might prevent the cell from NK-directed killing [51]. The striking observation of the unchanged death rates of virus-producing cells after CD8^+^ T cell depletion indeed challenges the view that CTL-mediated killing is effective during the late, productive stage of the cell [15], [16]. Here we have formally demonstrated that when CTL-mediated killing occurs early during the intracellular eclipse phase it can control HIV replication very efficiently, while remaining consistent with current observations on the up-slopes and down-slopes of the viral load observed during CD8^+^ T cell depletion experiments and antiretroviral treatment.

## Methods

### HIV dynamics model

For the mathematical analysis on the influence of clearing HIV-infected cells early or late during their viral life cycle, we devise a model of HIV dynamics that is based on standard models of within-host virus dynamics [52], [53]. In addition, we include an early stage of infected cells, that accounts for the eclipse phase during which cells do not produce virus yet [20], [54], [55]:
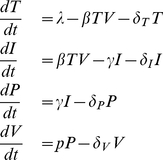
(1)


Here, non-infected CD4^+^ target cells 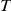 are produced at a rate of 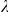 cells per day, die at a rate 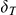 and can become infected by virus particles 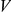 at a rate 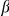 per day. An infected cell 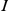 can either move through the viral eclipse phase with rate 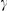 per day to become a virus-producing cell 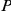, or die at a rate 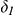 per day. Virus-producing cells 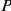 will die with the death rate 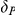. Viral particles 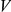 are produced at a rate 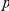 per day and are cleared at a rate 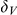. All parameters used for the analysis of the virus dynamics are given in Table 1. In *Results*, we vary the death rate of infected cells, 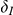, to account for early CTL-mediated killing during the viral eclipse phase. The potential effects of non-cytotoxic mechanisms mediated by CD8^+^ T cells are discussed below.

### Influence of cell death on virus production

To investigate the influence of cell death on virus production we calculate the expected duration of viral production by a cell that becomes infected with a virus. Increasing the death rate of infected cells, 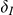, will reduce the fraction of cells that become virus-producing cells. The lifespan of virus-producing cells (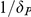) determines the amount of virus that is produced by a single cell. From Eq. 1, the average duration at which an infected cell will produce viral particles can be given by 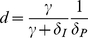
(2)


which suggests that changing 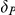 has a larger effect on virus production than changing 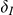.

### Viral load decay slopes

After the administration of antiretroviral drugs, the viral load declines exponentially during the first week and is typically related to the loss of virus-producing cells. Since the turnover of the virus is a fast process [56], we can set 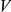 into a quasi-steady-state with the virus-producing cells 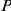 [57]. After the start of treatment, we assume that the infection rate 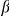 becomes 0 and the virus will decline as follows: 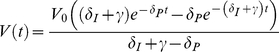
(3)


For any value of 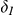, 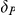 and 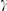, this allows us to calculate the exponential slope of the virus load decline at a specific time 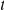 after drug treatment.

### CTL-mediated killing of infected cells

From the general scheme where an unbound CTL effector cell, 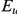, binds a target cell, 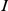, to form a complex, 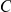, that after a time delivers cytotoxic killing and releases the effector cell, i.e., 
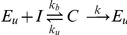
, one can make the total quasi-steady-state assumption (tQSSA), 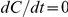, to obtain the following: 
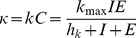
(4)


where 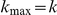 is now the maximal killing rate, and 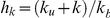 is a generalized Michaelis-Menten constant [54], [58], [59].

### Proliferation of CTL effector cells

CTL effector cells are stimulated by cells presenting viral antigen on their surface. Similar as for the killing of an infected cell, we can derive a general scheme where an unbound CTL effector cell, 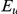, binds an antigen-presenting cell, 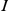, to form a complex, 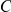, that after a time dissociates and causes the CTL effector cell to divide, i.e., 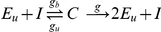. Making the total quasi-steady-state assumption (tQSSA), 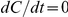, one obtains the following: 
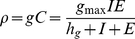
(5)


where 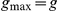 is now the maximal proliferation rate, and 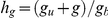 is a generalized Michaelis-Menten constant [54], [58], [59].

### Basic reproductive number, 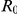


The basic reproductive number, 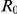, of a viral infection within a host is defined as the number of newly infected cells produced by one infected cell during its lifetime, assuming all other cells are susceptible [60]. From Eq. 1, it can be expressed as 
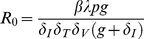
(6)


To calculate 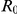 as a function of CTL killing, we substitute the death rate of infected cells during the non-productive stage of infection with 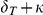. Assuming a constant CTL response, 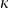 is simply the killing rate 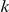. For the case where infected cells during the intracellular eclipse phase are killed by CTL effector cells, we compute 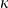 at the steady-state level of a chronic infection as a function of the saturation parameter of killing, 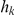.

### Non-cytotoxic mechanisms of CD8^+^ T cells

CD8^+^ T cells can also mediate non-cytotoxic effects on HIV-infected cells [42]. Mathematically, this can be described by a process function 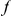 that will affect certain stages of the viral life cycle. Similar as in Muller et al. [2], we define 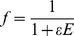
(7)


where CD8^+^ T cells 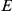 act as non-cytotoxic effector cells with efficacy 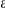. If CD8^+^ T cells reduce the number of new infections, the process function 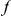 will reduce the infection rate 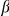, i.e., the total amount of new infections in Eq. 1 becomes 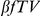. Non-cytotoxic mechanisms can also render newly infected cells non-infectious. This will affect the rate at which cells move through the eclipse phase, when the transition rate 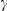 is affected by the process function, i.e., is given by 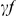. The infected cells that do not become virus-producing cells will instead render into target cells again with rate 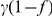.

The effects of a model with non-cytotoxic effects of CD8^+^ T cells on the virus dynamics are the same as shown in *Results*, except that the parameter region B in Fig. 2 is also a valid explanation for the invariant decline slope of the virus load during drug treatment experiments. Since infected cells are not cleared by CTL effector cells anymore, the death rate of infected cells 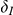, can be moderate and smaller than the rate at which cells move through the eclipse phase. Hence, the decay rate of viral load after drug treatment could in principle reflect the rate at which cells move through the eclipse phase, 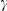, as it has been suggested by Klenerman et al. [20].
